# Mental health and substance use among international migrant workers in the Association of Southeast Asian Nations (ASEAN) countries: a systematic review and meta-analysis

**DOI:** 10.1080/16549716.2025.2548089

**Published:** 2025-09-03

**Authors:** Seo Ah Hong, Bang-On Thepthien, Doungjai Buntup, Mathuros Tipayamongkholgul

**Affiliations:** a Department of Public Health Administration, Faculty of Public Health, Mahidol University, Bangkok, Thailand; b ASEAN Institute for Health Development, Mahidol University, Nakhon Pathom, Thailand; c Department of Community Health, Faculty of Public Health, Mahidol University, Bangkok, Thailand; d Research Institute for Languages and Cultures of Asia, Mahidol University, Nakhon Pathom, Thailand

**Keywords:** Anxiety, depression, post-traumatic stress disorder, alcohol, tobacco, illicit drugs

## Abstract

Previous literature showed that mental health conditions and substance use are prevalent in international migrant workers due to acculturation stress. Given the rapid increase in labour migration within the Association of Southeast Asian Nations (ASEAN) countries, this study aimed to conduct a meta-analysis of the pooled prevalence of mental health conditions and substance use among international migrant workers in ASEAN countries and to identify associated factors. We searched MEDLINE, PubMed, Scopus, and ASEAN Citation Index (ACI) for articles published in English between January 2010 and October 2023. The included outcomes were mental health (depression, anxiety, and post-traumatic stress disorder (PTSD)) and substance use (alcohol, tobacco, and illicit drugs). Study quality was assessed using the Joanna Briggs Institute Qualitative Assessment and Review Instrument (JBI-QARI). Of 19 eligible studies, 18 articles (11 for depression, 5 for anxiety, 1 for PTSD, and 8 for substance use) were included in the meta-analysis. A significant number of studies included in this study targeted Myanmar migrant workers living in Thailand. The pooled prevalence was 34.77% for depression, 37.72% for anxiety, and 24.29% for substance use. Factors associated with mental disorders were younger age, being female, low education and income level, workers in construction and sex industry, while male gender, other substance use, and peer influence are associated with substance use. A high prevalence of mental disorders and substance use among international migrant workers warrants a collective effort by various parties to provide proactive support to prevent and manage mental health conditions and substance use in the ASEAN countries.

## Paper Context

- **Main findings:** The results of this meta-analysis showed that one in three international migrant workers in ASEAN countries have mental health conditions, and one in four have substance use.
- **Added knowledge:** Given cultural and geographic influences on mental health and substance use, this study provided regional estimates to inform better improvements in local health systems and services for mental health and substance use prevention and treatment.
- **Global health impact for policy and action:** This review highlights joint efforts by various parties to provide proactive support for the prevention, early detection, and treatment of mental health disorders and substance use among international migrant workers living in Association of Southeast Asian Nations (ASEAN) countries.

## Background

Globalisation has increased human mobility, particularly in the form of migration, in recent decades through improved interconnectivity between countries [1]. There were an estimated 281 million international migrants in 2020, and the proportion of international migrants relative to the global population is steadily increasing (from 2.8% in 1995 to 3.6%) [2]. By destination region, Asia not only sends out large numbers of emigrants but also hosts the most significant number of immigrants (85.6 million), alongside Europe (86.7 million) [2]. The regional cooperative association among Southeast Asian countries known as the Association of Southeast Asian Nations (ASEAN), established in 1967 may have accelerated labour migration within Southeast Asia [3]. Thailand and Malaysia emerged as top 20 destination countries in 2020, compared to 1995. Meanwhile, Indonesia, the Philippines, and Myanmar are among the top 20 countries with the largest number of emigrants in 2020 [2].

International migrant workers may be at high risk for mental health problems and substance **dependence [4,7–10]. They experience migration-related and acculturative stress during the acculturation process, as they must adapt to the sociocultural conditions of their host country, which may differ significantly from those of their home country [4,5]. In addition, they are usually engaged in 3D (dangerous, dirty, difficult) jobs and have a precarious position in the labour market. This leads to job insecurity and poor access to health services in the host country. These are associated with the vulnerability of migrant workers to mental health problems [6,7]. For these manual workers, substances are often used to improve work performance, improve career prospects, improve mood, and especially to make the user feel happy [11]. Social isolation due to unfamiliar living conditions, the absence of family and community support to discourage substance use, and the presence of a substance use culture in the host country increase the likelihood of their substance use [12]. A study comparing Mexicans living in the United States of America (USA) with Mexicans living in Mexico found that immigrants had increased opportunities for substance use due to increased availability and use of substances in the host country. However, this association was found for drugs but not alcohol [13]. Another systematic review among migrant and native adolescents in the European Union also supported that migrants were less likely to use alcohol, compared to natives. On the other hand, there were mixed results for tobacco and illicit drug use [14]. There are several systematic reviews of substance use among forced migrants, such as refugees or asylum seekers [15,16], migrant adolescents [14], or migrant women [17]. Yet, to the best of our knowledge, there are few systematic reviews of substance use among international migrant workers.

Given cultural and geographic influences on the mental health and substance use in the country of migration, regional estimates can better inform improvements in the region’s health systems and services for mental health and substance use prevention and treatment. However, regional data on the mental health and substance use of immigrant workers in the ASEAN countries are limited. Therefore, we performed a systematic review and meta-analysis of recent evidence from quantitative studies on mental health problems (anxiety, depression, and post-traumatic stress disorder (PTSD)) and substance use (alcohol, tobacco, and illicit drugs) and identified risk factors from qualitative analysis among international migrant workers in ASEAN countries. Consequently, this comprehensive systematic review and meta-analytic evidence identify knowledge gaps, provide evidence-based public health data on the magnitude of the problems, and offer insights for designing and planning policies and programs that promote the health and well-being of international migrant workers in ASEAN countries.

## Methods

### Protocol and registration

**Figure 1.**
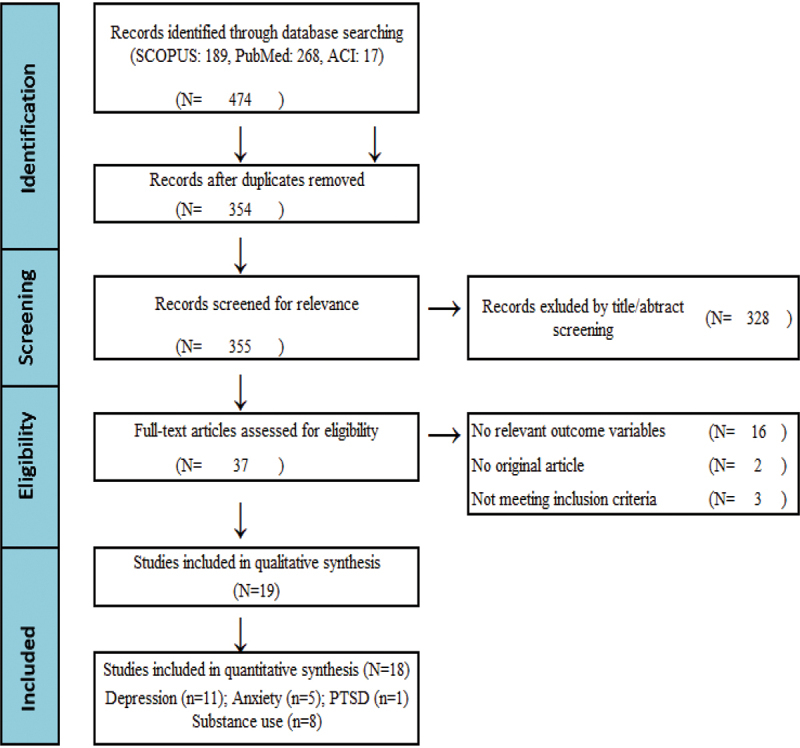
PRISMA (Preferred Reporting Items for Systematic Reviews and Meta-Analyses) flow diagram. Numbers of sources of evidence.

This review was conducted and written following Preferred Reporting Items for Systematic Reviews and Meta-analyses (PRISMA) guidelines [18] (Figure 1). The protocol of this study has been registered in the International Prospective Register of Systematic Reviews (PROSPERO) (registration number: CRD42023485566).

### Eligibility criteria

Papers that investigated the prevalence and related factors of mental health problems (depression, anxiety, or PTSD) and substance use (alcohol, tobacco, or illicit drugs) among international migrant workers. The Population, Exposure, Comparator, and Outcomes (PECO) framework [19] was used to determine the eligibility of original quantitative studies in peer-reviewed journals. Studies were selected based on PO within the PECO framework, considering the research objectives. The population refers to international migrant workers aged 15 years or older who were working in a foreign country other than their country of origin, and the countries in which they were working outside their country of origin included 11 ASEAN countries (Brunei Darussalam, Cambodia, Laos, Indonesia, Malaysia, Myanmar, the Philippines, Singapore, Thailand, Timor-Leste, and Vietnam). This study primarily focused on international economic migrant workers who migrated for economic opportunity and sought to understand their mental health and substance use, because international migrant workers, asylum seekers, and refugees face unique, complex, multifactorial mental health challenges, as their legal status and relationship to their country of residence also vary considerably. The outcomes refer to mental health conditions (depression, anxiety, or PTSD) and substance use (alcohol, tobacco, or illicit drugs). Articles that used observational quantitative study design, including mixed-methods study designs, and were published in English from January 2010 to October 2023 were included. Papers published in languages other than English, papers published before 2010, or review papers were excluded. Publications targeting internal migrant workers moving within the same country, refugees, or asylum seekers were not included.

### Search strategy and selection process

Database searches were systematically conducted on MEDLINE, PubMed, Scopus, and the ASEAN Citation Index (ACI) using the keywords and search terms (Table 1). Additionally, when we did not receive an article or quantitative research information was inadequate, we attempted to obtain the full text by contacting the corresponding author of the paper via email. When two studies with the same participants were included, only papers where the focus of the outcome variable was the same as our study focus were included in the meta-analysis. Studies with low-quality assessment scores were not excluded from the review to identify the knowledge gaps in the studies; however, studies presenting combined prevalence data for more than one disorder (e.g. common mental health disorders) were excluded. Data were extracted from the selected papers using EndNote X20 (Clarivate Analytics, USA) to remove duplicate studies and retrieve relevant studies from the selected databases for the systematic review.

**Table 1.** Population, Exposure, Comparator, and Outcomes (PECO) framework and searching strategies.

The Population, Exposure, Comparator, and Outcomes (PECO) framework (19) • Population: international migrant workers aged 15 years or older who were working in a foreign country other than their country of origin, and the countries in which they were working outside their country of origin included 11 ASEAN countries (Brunei Darussalam, Cambodia, Laos, Indonesia, Malaysia, Myanmar, the Philippines, Singapore, Thailand, Timor-Leste, and Vietnam • Exposure: No • Comparator: No • Outcomes: mental health conditions (depression, anxiety, or PTSD) and substance use (alcohol, tobacco, or illicit drugs) **The following keywords** *For outcome variables* (1) ‘Mental health’ OR ‘Mental illness’ OR ‘Mental disorder’ OR ‘Psychiatric illness’ OR ‘Psychiatric burden’ (2) ‘Anxiety’ OR ‘Anxiety disorder’ (3) ‘Depression’ OR ‘Depressive disorder’ OR ‘Depressive symptoms’ OR ‘Major depressive disorder’ (4) ‘Posttraumatic stress disorder’ OR ‘PTSD symptoms’ (5) ‘Substance’ OR ‘Substance use’ OR ‘Substance abuse’ OR ‘Substance disorder’ OR ‘Illicit drugs’ OR ‘drugs’ (6) ‘Alcohol use’ OR ‘Alcohol abuse’ OR ‘Alcohol dependence’ (7) ‘Tobacco’ OR ‘Smoking’ OR ‘Smokeless tobacco’ *For population* (8) ‘Migrant workers’ OR ‘Migrants’ OR ‘Immigrants’ (9) ‘Brunei’ OR ‘Cambodia’ OR ‘Laos PDR’ OR ‘Laos’ OR ‘Malaysia’ OR ‘Myanmar’ OR ‘Burma’ OR ‘Indonesia’ OR ‘Philippines’ OR ‘Timor-Leste’ OR ‘Thailand’ OR ‘Singapore’ OR ‘Vietnam’ **Combined terms**: 1 AND 8 AND 9; 2 AND 8 AND 9; 3 AND 8 AND 9; 4 AND 8 AND 9; 5 AND 8 AND 9; 6 AND 8 AND 9; 7 AND 8 AND 9.

#### Quality assessment

Both title and abstract screening were carried out, followed by an analysis of the full-text articles. The Joanna Briggs Institute (JBI) critical appraisal checklist for studies reporting prevalence data (2020) was used to evaluate the quality of the papers [20]. The risk of bias in the included studies was independently assessed by two reviewers (SAH and DB), and any disagreements were resolved through discussion between the reviewers. Quality assessment questions and scores for evaluating each paper were included and are presented in Table 2.

**Table 2.** Quality assessment results of the included studies.

No.	Authors (Year)[ref]	1. Was the sample frame appropriate to address the target population?	2. Were study participants sampled in an appropriate way?	3. Was the sample size adequate?	4. Were the study subjects and the setting described in detail?	5. Was the data analysis conducted with sufficient coverage of the identified sample?	6. Were valid methods used for the identification of the condition?	7. Was the condition measured in a standard, reliable way for all participants?	8. Was there appropriate statistical analysis?	9. Was the response rate adequate, and if not, was the low response rate managed appropriately?	TOTAL SCORE	
1	Gaitan D et al., (2018) [21]	1	1	0	1	1	1	1	1	1	8	High
2	Poe NE et al., (2022) [22]	1	1	1	1	1	1	1	1	1	9	High
3	Chamroen P et al., (2020) [23]	1	1	1	1	1	1	1	1	0	8	High
4	Guadamuz TE et al., (2018) [24]	1	1	0	1	1	1	1	1	1	8	High
5	Saw JE et al., (2021) [25]	1	1	1	1	1	1	1	1	1	9	High
6	Aung TN et al., (2019) [26]	0	1	1	1	1	1	1	1	1	8	High
7	Decker MR et al., (2021) [27]	1	0	0	1	1	1	1	1	1	7	Mod
8	Aurpibul L et al., (2022) [28]	1	1	1	1	1	1	not certain	1	not certain	7	Mod
9	Charoenca N et al., (2021) [29]	not certain	not certain	1	1	1	1	1	1	1	7	Mod
10	Htay MNN et al., (2020) [30]	1	0	1	1	1	1	1	1	1	8	High
11	Siregar PP et al., (2021) [31]	1	1	1	1	1	1	1	1	1	9	High
12	Pocock NS et al., (2018) [32]	1	1	1	1	1	1	1	1	1	9	High
13	Meyer SR et al., (2015) [33]	1	1	1	1	1	1	1	1	1	9	High
14	Htin K et al., (2014) [34]	1	1	1	1	1	1	1	1	1	9	High
15	Jaichuang S et al., (2012) [35]	0	0	1	1	1	not certain	not certain	1	not certain	4	Low
16	Aung TNN et al., (2020) [36]	1	1	1	1	1	0	1	1	1	8	High
17	Racal SJ et al.,(2020) [37]	1	1	1	1	1	1	1	1	1	9	High
18	Kesornsri S et al., (2019) [38]	1	1	1	1	1	1	1	1	1	9	High
19	Aung H and Perngparn U (2014) [39]	1	1	0	1	0	not certain	not certain	1	not certain	4	Low

#### Statistical analysis

All data analyses were performed using STATA® (version 18; College Station, TX, USA: Stata Corp). Prevalence estimates of mental health condition variables (e.g. depression, anxiety, PTSD, and substance use variables (e.g. alcohol, tobacco, and illicit drugs) were calculated with 95% confidence intervals (CI) using forest plots. To account for between-study heterogeneity, we used random-effects meta-analyses using the DerSimonian and Laird estimator based on inverse variance weights. The presence of between-study heterogeneity was tested using the *I*^2^ statistic (*I*^2^ ≥75%) [40]. Identification of publication bias was detected using Egger’s test. In addition, associated factors were synthesised narratively according to outcome variables. Sensitivity analyses were conducted according to study quality, country of origin, host country, and screening tools in studies assessing depression only.

#### Ethical considerations

This systematic review study does not require ethical approval because it does not include data from individuals, and individuals are not identifiable.

## Result

### Study selection and quality assessment

Electronic database searching identified a total of 474 articles. After removing duplicate publications, the titles and abstracts were screened and selected. A total of thirty-seven studies were included after a full-text review. Of the thirty-seven studies, only nineteen articles received quality assessment. Of them, eighteen studies (depression (*n* = 11), anxiety (*n* = 5), PTSD (*n* = 1), substance use (*n* = 8) were employed in the quantitative meta-analysis. A PRISMA flow chart presented in Figure 1 illustrates data extraction performed following the Quality of Reporting of Meta-analyses Guidelines.

Table 2 presents a summary of study quality, as assessed using the JBI critical appraisal checklist. Of the 19 studies, 14 could be regarded as high quality [21–25,30–34], three as moderate quality [27–29], and two as low quality [35,39].

### Prevalence of mental health problems

Twelve studies were included for mental health problems, such as depression, anxiety, and PTSD (Table 3). Among them, four studies assessed both depression and anxiety [25,27,31,38], and only one study assessed all three outcomes [32]. All the studies were cross-sectional and were published between 2018 and 2022. Almost all studies have been conducted in Thailand (*n* = 8; one study was conducted in both Cambodia and Thailand), followed by Malaysia (*n* = 2) and Singapore (*n* = 1). Most of them consisted of study populations that involved migrant workers with work permits (*n* = 8). In contrast, only two studies included both documented and undocumented migrant workers, and one study did not specify it. Two studies targeted migrant workers working in the sex industry [27,33], and one study in trafficked fishermen using post-trafficking services [32], and one living with HIV [28].

**Table 3.** Summary of characteristics of studies included in the analysis of mental disorders.

Author (Year)	Country of destination	Country of origin	Study design	Participants	Sampling	Document status	Male %	age group	Measurement tool	Outcome %
Meyer SR et al., (2015) [33]	Thailand	Myanmar	Mixed-method (Cross-sectional)	197 agriculture,252 factory, 123 sex industry	Respondent-driven	Both	33.5	18–34	HSCL-25	Depression = N.R. Anxiety = N.R.
Pocock NS et al., (2018) [32]	Thailand and Cambodia	Cambodia, Myanmar, Thailand	Mixed-method (Cross-sectional)	275 trafficked fishermen using post-trafficking services	Purposive/snowball	Both	100	12–55	HSCL-25 for depression and anxiety (>1.75 and >1.625, respectively) HTQ for PTSD	Depression = 54.2% Anxiety = 44.7% PTSD = 39.3%
Aung TN et al., (2019) [26]	Thailand	Myanmar	Cross-sectional	414 migrant workers	Convenience	Registered	55.8	18–60	PHQ 2 and 9 (≥5)	Depression = 13.0%
Kesornsri S et al., (2019) [38]	Thailand	Myanmar	Cross-sectional	445 migrant workers	PPS	Registered	55.5	18–60	HSCL-25 (≥1.75)	Depression = 9.0% Anxiety = 8.1%
Chamroen P et al., (2020) [23]	Thailand	Cambodia	Cross-sectional	1,211 migrant workers	Multistage random sampling	Registered	50.4	N.R.	CES-D	Depression = 69.7%
Htay MNN et al., (2020) [30]	Malaysia	Myanmar	Cross-sectional	192 migrant workers	Convenience	Registered	73.4	≥18	WHO-5 (≤28)	Depression = 70.8%
Racal SJ et al.,(2020) [37]	Thailand	Myanmar	Cross-sectional	186 young migrant workers	PPS	Registered	60	18–24	CES-D (≥16)	Depression = 8.6%
Decker MR et al., (2021) [27]	Thailand	Myanmar	Mixed-method (Cross-sectional)	Women in the Sex Industry	Respondent-driven sampling	N.R.	0	18–32	HSCL-25 (>1.75)	Depression = 87.5% Anxiety = 75.8%
Saw JE et al., (2021) [25]	Singapore	Bangladesh, India, Others	Cross-sectional	1011 migrant workers	Convenience	Registered	100	21–60	DASS-21 (≥10 for depression and ≥8 for anxiety)	Depression = 21.3% Anxiety = 17.5%
Siregar PP et al., (2021) [31]	Malaysia	Indonesia	Cross-sectional	589 migrant workers	Simple random	Registered	0	18–45	DASS-21 (≥10 for depression and ≥8 for anxiety	Depression = 23.6% Anxiety = 52.3%
Aurpibul L et al., (2022) [28]	Thailand	Majority (Myanmar Shan)	Cross-sectional	316 migrant workers living with HIV	Convenience	Registered	35.1	≥18	PHQ-9 (≥5)	Depression = 19.0%
Poe NE et al., (2022) [22]	Thailand	Myanmar	Cross-sectional	402 migrant workers	Simple random sampling	Both	42.0	≥18	PHQ-2 (≥1)	Depression = 24.1%

CES-D: The Center for Epidemiology Studies Depression Scale DASS-21: The Depression, Anxiety, and Stress Scale 21 *HSCL-25*: The Hopkins Symptom Check List-25 HTQ: Harvard Trauma Questionnaire PHQ-2: The Patient Health Questionnaire-2 PHQ-9: The Patient Health Questionnaire-9.

PPS: Probability Proportion to Size SRQ: WHO’s Self Reporting Questionnaire TMHI-15: Thai Mental Health Indicator-15.

N.R.: Not Reported.

**Figure 2.**
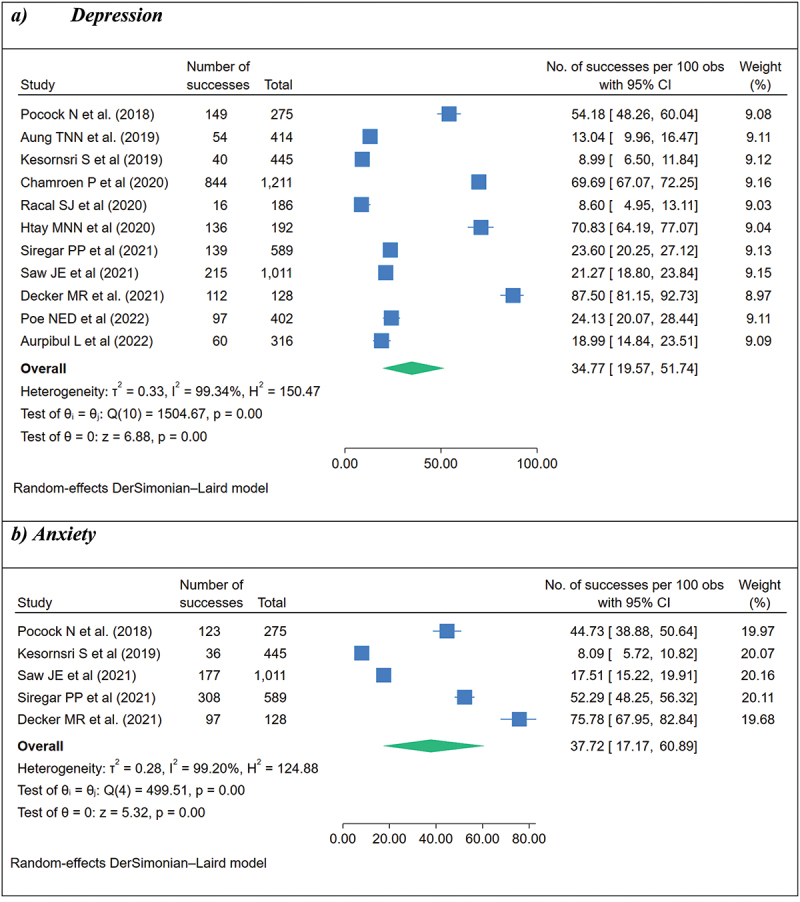
Pooled prevalence of depression (a) and anxiety (b) among migrant workers working in the eleven ASEAN member countries.

To assess the prevalence of depression, various tools were used, including the HSCL-25 (*n* = 3), CES-D (*n* = 2), DASS-21 (*n* = 2), PHQ-9 (*n* = 2), PHQ-2 (*n* = 1), and WHO-5 (*n* = 1). There were 5,169 participants included from the eleven studies, with a sample size ranging from 128 to 1,211. Regarding gender composition, eight studies included male and female participants. While another four studies had more male participants (ranging from 50.4% to 73.4%), two studies had more female participants. In addition, four studies had participants of a single gender (two studies for female participants only and two for male participants only). Prevalence of depression ranged from 8.6% to 87.5% (Figure 2). An analysis of the eleven studies revealed a pooled prevalence of 34.77% (95% CI = 19.57–51.74), with high heterogeneity (*p* < 0.001, I^2^ = 99.34%).

Egger’s test: Beta = 12.39 (SE = 10.796), *p* = 0.2511 for depression and beta = 20.34 (SE = 10.974), *p* = 0.0638 for anxiety.

For anxiety, five studies measuring anxiety used the DASS-21 (*n* = 2) and HSCL-25 (*n* = 3) in cross-sectional studies, which were conducted in Thailand (*n* = 2), Thailand and Cambodia (*n* = 1), Singapore (*n* = 1), and Malaysia (*n* = 1), and were published between 2015 and 2021. The prevalence ranged from 8.1% to 75.8% and the pooled prevalence was 37.72% (95% CI = 17.17–60.89) with high heterogeneity (*p* < 0.001, I^2^ = 99.20%).

For PTSD, only one study was identified [32], in which migrant workers were recruited at post-trafficking services in Thailand and Cambodia. The prevalence was 39.4% as measured by the Harvard Trauma Questionnaire (HTQ).

### Factors associated with mental health outcomes

Table 4 shows factors associated with mental health outcomes. For depression, of personal factors, most studies reported no associations, while a few studies reported that factors associated with depression are younger age [31], being female [28,38], not being married [25], low education [23], high income [25], a higher number of children [41], and longer years spent in host country [30].

**Table 4.** Summary of factors associated with mental disorders.

	Depression	Anxiety
**Personal factors**		
Young age	NA [23,25,28,30,41]: + [31]:	NA [25]: + [31]:
Country of origin	NA [25]:	NA [25]:
Female gender	+ [28,38]: NA [23,30,41]:	
Not being married	+ [25]: NA [28,30]:	NA [25]:
Low education	+ [23]: NA [25,28,30,41]:	
High monthly income	+ [23]: NA [25]:	NA [25]:
Health insurance	NA [30]:	
Religiosity score	NA [31]:	NA [31]:
Years spent in the host country	+ [30]: NA [25,31,41]:	NA [25,31]:
Number of children	+ [41]:	
**Occupational factors**		
Occupation (construction vs. agriculture/service)	+ [23]:	
Length of hours of work a day	NA [31]:	NA [31]:
Housing tenure (staying in the employer’s accommodation)	+ [23]:	
Financial help from the employer if they have a physical illness	- [30]:	
**Socio-environmental factors**		
Staying alone	+ [23]:	
Experience of discrimination or exploitation	+ [41]:	
Sexual assault and abuse	+: in male [33]	+: both [33]
Daily hassles and stressors	+: in female [33]	+: both [33]
Barriers to exit	+: in female [33]	
**Physical and psychological factors**		
High stress	+ [23]:	
Security stressors in women	+ [33]:	
Low quality of life	+ [23]:	
Reproductive problems	+ [31]:	+ [31]:
Self-rated health	- [25]:	- [25]:
**COVID-19-related factors**		
Tested positive for COVID-19	- [25]:	+ [25]:
Fear of losing a job during the COVID-19 outbreak	+ [25,42,43]:	+ [25]:
Exposure to COVID-19 rumors	+ [25]:	+ [25]:
Worries about susceptibility to COVID-19/Health fears during COVID-19 outbreak	+ [25,42]:	+ [25]:
Worries about family members’ safety from COVID-19 Infection	+ [42]:	
Fear of detention and subsequent waves	+ [42]:	
Movement restriction during the COVID-19 outbreak	+ [25,42]:	+ [25]:

+: Positive association -: Inverse association N.A.: No Association.

[] indicates the reference number of articles.

Most studies recruited unskilled migrant workers. Among occupational factors, a study revealed that workers in construction, compared to agriculture/service, and those staying in the employer’s accommodation, compared to those renting, were more likely to suffer from depression [23]. When staying in the employer’s accommodation [23] and having no financial help from the employer if they have a physical illness [30] were associated with depression. In terms of socio-environmental factors, experience of discrimination or exploitation [41], staying alone compared to staying with family/relatives/friends [23] were associated with depression. Another study among migrant workers on the Thailand – Myanmar border reported that sexual assault and abuse were associated with depression only in male migrant workers, while daily hassles and stressors and barriers to exit were associated in females but not in males [33]. Furthermore, psychological factors, such as high stress [23], security stress [33], and low quality of life [23], were associated with depression. Reproductive problems [31] were positively associated with depression, while self-rated health [25] was inversely associated with depression. Several studies in Singapore and Thailand have investigated the associations during the COVID-19 era. Being tested positive for COVID-19 [25], fear of losing job during COVID-19 outbreak [25,42], exposure to COVID-19 rumours [25], health fears during COVID-19 outbreak [25,42], movement restriction during COVID-19 outbreak [25,42], worries about family members’ safety from COVID-19 infection, and fear of detention and subsequent waves [42] were associated with depression.

For anxiety, young age was the only personal factor found to be associated [31], while no associations with other factors were reported. In terms of socio-environmental factors, sexual assault and abuse, and daily hassles and stressors were associated with anxiety in both male and female [30]. Furthermore, psychological factors, reproductive problems [31], and self-rated health [25] were positively associated with anxiety. Studies in Singapore and Thailand during the COVID-19 era revealed that being tested positive for COVID-19 [25], fear of losing a job during the COVID-19 outbreak [25,42], exposure to COVID-19 rumours [25], worries about susceptibility to COVID-19/health fears during the COVID-19 outbreak [25,42]. Movement restriction during the COVID-19 outbreak [25,42] was associated with anxiety.

### Prevalence of substance use

**Figure 3.**
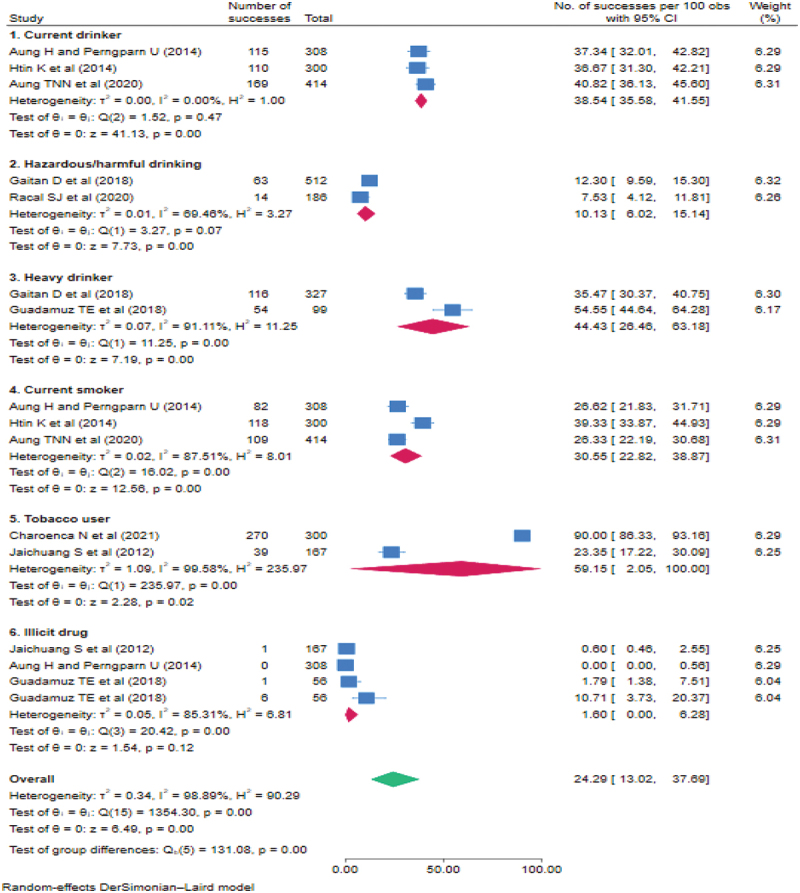
Pooled prevalence of types of alcohol, tobacco, and illicit drugs among migrant workers working in the eleven ASEAN member countries.

Table 5 and Figure 3 show the study characteristics of included studies and the prevalence of substance use. For alcohol consumption, all the included articles (*n* = 6) were cross-sectional studies, published between 2014 and 2022. Most studies targeted semi-skilled or low-skilled Myanmar workers with work permits in Thailand. The pooled prevalence of alcohol consumption was 30.52% (95% CI = 19.07–43.34, I^2^ = 97.37%). When stratified by types of alcohol consumption, the pooled prevalence of current alcohol drinking and hazardous/harmful drinking was 38.54% (95% CI = 35.58–41.55, I^2^ = 0.00%) and 10.13% (95% CI = 6.02–15.14, I^2^ = 69.46%), respectively, with low heterogeneity; heavy drinking had high heterogeneity at 44.43% (95% CI = 26.46–63.18, I^2^ = 91.11%).

Egger’s test: Beta = −6.49 (SE = 5.645), *p* = 0.2500 for substance use; beta = 6.92 (SE = 8.660), *p* = 0.4241 for alcohol and beta = −10.61 (SE = 33.907), *p* = 0.7544 for tobacco.

**Table 5.** Summary of characteristics of studies included in the analysis of substance abuse.

Author (Year)	Country of destination	Country of origin	Study design	Participants	Sampling	Document status	Male %	age group	Measurement tool	Outcome %
Jaichuang S et al., (2012) [35]	Thailand	Laos PDR	Mixed-method (Cross-sectional)	300 migrant workers	Convenience	Both	46.7	≥15	Not specified	Tobacco use = 23.4% Methamphethamine = 0.6%
Aung H and Perngparn U (2014) [39]	Thailand	Myanmar	Cross-sectional	308 migrant workers	Purposive/snowball	Both	53.9	≥18	Not specified	Currently drinking = 37.3% Currently smoking = 26.6% Methamphethamine = 0%
Htin K et al., (2014) [34]	Thailand	Myanmar	Cross-sectional	300 laborers	PPS	Both	56.0	18–24	Drinking in the past 30 days Smoking in the past 30 days	Currently drinking = 36.7% Currently smoking = 39.3% Alcohol+Smoking = 27.3%
Gaitan D et al., (2018) [21]	Thailand	Myanmar	Cross-sectional	512 migrant workers	Convenience	N.R.	100	≥15	HHD: Using the AUDIT, an AUDIT cutoff score of 8 or higher; Heavy drinking: drinking at least 60 grams or more of pure alcohol on at least one occasion in the past seven days (WHO; 2014)	HHD = 12.3% Heavy drinking monthly = 35%
Guadamuz TE et al., (2018) [24]	Thailand	Cambodia, Lao, Vietnam, Myanmar	Cross-sectional	87 migrant and 125 thai male sex workers (*n* = 212)	Not specified	N.R.	100	18–24	Heavy drinking: Consuming 10+ alcohol beverages per day and having consumed alcohol beverages for 10+ days in the past 30 days (Center for Behavioral Health Statistics and Quality, USA; 2015).	Heavy drinking monthly = 54.5% Methamphethamine (90 days) = 1.8% Crystal Meth/Ice/Sketch (90 days) = 10.7%
Aung TN et al., (2020) [36]	Thailand	Myanmar	Cross-sectional	414 migrant workers	Stratified systematic	Registered	55.8	18–60	Consuming any type of alcoholic drinks (spirit, beer, wine) regularly or irregularly during the previous year Smoking any tobacco products (cigarettes, tobacco, or cheroot) either on some days or every day	Currently drinking = 40.8% Currently smoking = 26.3%
Racal SJ et al., (2020) [37]	Thailand	Myanmar	Cross-sectional	186 young migrant workers	PPS	Registered	54.8	18–24	HHD: Using the AUDIT, an AUDIT cutoff score of 8 or higher;	HHD = 7.5%
Charoenca N et al., (2021) [29]	Thailand	Myanmar	Cross-sectional	300 migrant factory workers in the seafood industry	Purposive	N.R.	94.3	N.R.	Using manufactured or hand-rolled cigarettes or smokeless tobacco in the 30 days	Tobacco use = 90.0%

AUDIT: The Alcohol Use Disorders Identification Test HHD; Hazardous/harmful drinking.

PPS: Probability Proportion to Size N.R. = Not Reported.

For current smoking, five articles included were cross-sectional studies (*n* = 5) and published in Thailand between 2012 and 2021. All studies were conducted in Thailand, with four studies specifically targeting Myanmar migrant workers. The pooled prevalence of tobacco use was 41.76% (95% CI = 17.18–68.76, I^2^ = 99.13%). When stratified by types of tobacco use, the pooled prevalence of current smoking and tobacco use was 30.55% (95% CI = 22.82–38.87, I^2^ = 87.51%) and 59.15% (95% CI = 2.05–100, I^2^ = 99.58%), respectively.

For illicit drugs, three studies assessed methamphetamine, and the prevalence ranged from 0% to 1.8%, while one study reported taking 10.7% of migrant workers had ever taken Crystal Meth/Ice/Sketch in the last 90 days. The pooled prevalence of illicit drugs was 1.60% (95% CI = 0–6.28, I^2^ = 85.31%).

One study revealed that 27.3% reported using both tobacco and alcohol, and 21% reported using three substances (smoking, alcohol, and/or betel quid chewing) [34]. When all substances were combined in our study, the pooled prevalence of total substance use was 24.29% (95% CI = 13.02–37.69, I^2^ = 98.89%).

### Factors associated with substance use

As shown in Table 6, factors associated with current alcohol drinking were ethnicity (Myanmar vs. Shan) [36], male gender [36,37,39], years spent in host country [37], high monthly income [36,39], history of drinking in country of origin [37] and tobacco use [39]. Regarding hazardous/harmful drinking, only one study among Myanmar migrant workers in Thailand reported that older age, being married, years spent in the host country, frequent visits home in Myanmar, and living in townships were associated [21]. A study in Thailand revealed that current stimulant use, 15+ persons of client partners, and age at which they first consumed alcohol were associated with heavy drinking among 212 migrant and non-migrant male sex workers in Bangkok and Pattaya, Thailand [24].

**Table 6.** Summary of factors associated with substance use.

	Alcohol		Currently smoking
	Hazardous/harmful drinking (HHD)	Currently drinking	
**Personal factors**			
Age	+ [40]: NA [41]:	NA [42]:	NA [42]:
Ethnicity (Myanmar vs. Shan)		+ [42]:	NA [42]:
Male gender	NA [41]:	+ [34,39,42]:	+ [42,43]:
Married	+ [40]:		
Low education	NA [40,41]:	NA [42]:	NA [42]:
Years spent in the host country	+ [40]:	+ [34]:	
Frequent visits home in Myanmar	+ [40]:		
High monthly income	NA [40]:	+ [39,42]:	NA [42]:
Registration status			
Earlier initiation age of alcohol consumption	NA [40]: + [37,41]:		
History of drinking in the country of origin		+ [34]:	
Family history of alcohol use		NA [34]:	
Alcohol use			+ [39]:
Current stimulant use	+ [41]:		
**Occupational factors**			
Occupation (construction vs. agriculture/service)	NA [40]:		
Length of hours of work a day	NA [40]:		NA [43]:
**Socio-environmental factors**			
Area of residence (townships)	+ [40]:		
Higher number of people in residence	NA [40]:		
Number of client partners (15+)	+ [41]:		
Having friends who smoke			+ [43]:
Having friends who smoke among the 10 closest friends			NA [43]:
A friend persuades me to smoke			NA [43]:
Having people who smoke in the family			NA [43]:
**Physical and psychological factors**			
Perceived stress			NA [43]:
Depressive symptoms		NA [34]:	
Acculturative stress		NA [34]:	
Self-esteem		NA [34]:	
High separation		NA [42]:	NA [42]:
High integration		NA [42]:	NA [42]:
High assimilation		NA [42]:	NA [42]:
Low marginalization		NA [42]:	+ [42]:

+: Positive Association -: Inverse Association NA: No Association.

[] indicates the reference number of articles.

Smoking was common among male migrants. A study among Myanmar male migrant factory workers in the seafood industry in Thailand showed that 90% of male migrants smoked, and many of their friends smoked [29]. However, there was no association of smoking with personal factors, such as age, ethnicity, education, income, and registration status in those studies. Another study of Myanmar migrant workers in Chiang Mai, Thailand, showed an inverse association between a marginalisation score and current smoking [36]. A study among 300 young adult Myanmar labourers (aged 18–24 years) in Mae Sot District, Tak Province, Thailand [34] showed that most people who smoke (83.1%) reported to have started smoking before the age of 20 years, to have smoked 7 days per week (78.8%) and not to cease smoking within the next 6 months (65.3%). Another study among migrant workers of the Thai-Laos border in Thailand also showed migrants used various kinds of substances, such as tobacco, energy drinks, coffee, and methamphetamine, and those who receive information from community radios/news are associated with smoking [35]. Meanwhile, one study revealed that the poly-substance use behaviours were higher in males compared to females [34].

## Discussion

Our systematic review and meta-analysis of the literature attempted to estimate the burden of mental disorders (anxiety, depression, and PTSD) and substance use (alcohol, tobacco, and illicit drugs) and to identify associated factors among international migrant workers within the eleven ASEAN countries. Despite considerable heterogeneity, around one in three international migrant workers has mental health problems (34.77% for depression and 37.72% for anxiety) and one in four uses substances (24.29%). Younger age and exposure to occupational and physical/psychological deprivation, including during COVID-19, were found to be associated with depression and anxiety. Substance use has been associated with male gender, concurrent use of other drugs, and peer influence, but research on this is lacking.

In total, while all eight studies included in the meta-analysis on substance use were done only in Thailand, twelve articles on mental disorders were conducted in four countries with nine studies in Thailand (including one study conducted in both Thailand and Cambodia) and the remainder in Malaysia and Singapore. One of the reasons for the small number of studies conducted in countries other than Thailand is that some studies in Singapore [44] and in Malaysia [41] that did not differentiate between depression and anxiety symptoms were not included in the meta-analysis. In addition, the reason why much research has been done on immigrants in these three countries is that Malaysia and Thailand, upper-middle-income countries, and Singapore, a high-income country, are major destinations for migrant workers from other lower-middle-income countries. Malaysia and Singapore have many immigrants from bordering countries (e.g. Thailand), as well as Myanmar, the Philippines, Bangladesh, and Indonesia. On the other hand, since Thailand shares borders with Myanmar, Cambodia, and Lao PDR, most immigrants come from these neighbouring countries [2]. Of the studies conducted in Thailand, most targeted Myanmar migrants. Migration from Myanmar to Thailand is one of the top 20 international long-term cross-border corridors, accounting for a significant share of international migration in 2020 [2], which has led to considerable research on Myanmar migrants in Thailand.

In terms of the prevalence of mental health conditions, our meta-analysis showed a high pooled prevalence of depression (34.77%) and anxiety (37.72%). Compared to other meta-analyses reporting prevalence rates among international migrants worldwide, the prevalence of mental disorders among migrant workers in the ASEAN region is similar. A meta-analysis result in the global African migrant population, including refugees and asylum seekers, found that the pooled prevalence was 34.60% (95%CI = 26.30–43.00) for anxiety, 33.20% (95%CI = 27.70–38.37) for depression, and 37.9% (95%CI = 23.5–52.4) for PTSD [45]. Another review study among 44,365 migrant workers in 17 countries found that the prevalence of depression and anxiety was 38.99% (95% CI = 27–51) and 27.31% (95% CI = 6–58), respectively [46]. Meanwhile, our meta-analyses of prevalence showed very high *I*^2^ values. Differences in prevalence estimates were large between studies, ranging from 8.60% to 87.50% for depression and 8.09% to 75.78% for anxiety. This finding could be due to several factors, including sociodemographic profiles or methodological differences. Sensitivity analysis showed a significant difference in prevalence estimates between the assessment tools used. While two studies [25,31] using the DASS-21 showed a prevalence of 22.2% with a low I2 value (15.32%), those of the other screening instruments showed a high I2 value (78.92% for PHQ-9 and approximately 99.5% for CES-D and HSCL-25). Usage of various assessment tools to measure mental disorders, such as HSCL-25, CES-D, DASS-21, and PHQ-9, may lead to a variation in the prevalence of mental disorders. Since all studies used screening tools instead of diagnostic tools, the prevalence estimates may be subject to under- or over-estimation. Additionally, the prevalence estimates varied by study quality. Prevalence of depression was lower in high-quality studies (30.7%, 95% CI = 15.2–48.9) than in moderate-quality studies (54.3%, 95% CI = 0.6–100). In studies that included only documented migrant workers, the pooled prevalence was lower (27.3%, 95% CI = 11.3–47.1) than in studies that included both documented and undocumented migrant workers (38.6%, 95% CI = 12.5–68.7). This highlights the need for caution in interpreting our pooled estimates. Regarding PTSD, Pocock et al. [32], who published the only study reporting PTSD prevalence, found that migrant workers attending post-trafficking services in Cambodia and Thailand had a high prevalence of PTSD (39.4%) due to extreme adversity conditions during the migration process. More studies are needed to capture an accurate estimate of PTSD and anxiety disorders in migrant workers.

As of April 2023, the number of immigrants in Thailand is estimated to be between 4 and 5 million, of which 2.5 million hold regular status [47]. Since some migrants were reluctant to disclose their visa status, which may be indicative of their vulnerability or irregular status, the target group in most studies included in the review for mental health was documented migrants. Little research has studied undocumented migrants’ mental health [42]. A study in the USA among Hispanic communities showed no significant difference in the prevalence of depression and anxiety by immigration status. However, undocumented migrants are less likely to use antidepressants [48]. On the other hand, some studies in Europe reported poor mental health among migrants with undocumented status [49–52], consistent with our results that undocumented migrants had a higher rate compared with their counterparts. It may be explained by the fact that among workers without a written employment contract, experiences of being paid less than the national hourly minimum wage and experiences of being insulted and/or threatened are more likely to increase depression [52]. Furthermore, most research included in our review studied semi- or low-skilled workers. Migrant workers with a history of trafficking who work in the sex industry tend to suffer from more mental disorders (depression: 87.5% and anxiety: 75.8%) [27], compared to other migrant workers. Since legal labour protections such as occupational health and safety standards, including minimum wages and maximum work hours, do not extend to agriculture or the sex industry, more efforts are needed to enhance mental health among migrant workers in agriculture or the sex industry. Furthermore, in studies of migrant workers, it is challenging to use robust sampling because migrant populations, especially undocumented immigrants, are often reluctant to reveal their identities. Thus, because many studies used convenience sampling for small sample sizes and unrepresentative samples of migrant workers, there are limitations in generalising the results of this study. More studies on mental health are needed with robust sampling methods to represent international migrant workers in the ASEAN member countries that are the destination countries for these migrants.

Our review identified a variety of factors associated with mental disorders, including personal, psychological, occupational, socio-environmental, and COVID-19-related factors. Regarding personal factors, although a few studies have supported significant associations with factors such as age, gender, and longer duration of stay, most studies have not shown positive associations between personal factors and mental health outcomes. Regarding occupational, socio-environmental, and psychological factors, we found that only a limited number of studies investigated the associations with mental health. A systematic review [46] identified an association between mental illness and occupational stressors, such as poor working conditions, wage and benefit issues, and workplace abuse among migrant workers. Meyer et al. [33] found that migrant workers with stressors, such as experience of discrimination or exploitation, daily hassles, and sexual assault and abuse, reported a higher level of depression and anxiety, and types of stressors affecting mental disorders are different between men and women and between occupations. This may be supported by our study reporting that migrant workers working in construction or the sex industry have a significant association with depression. A few studies investigated in the COVID-19 era were also included in this current study. Some COVID-19-related factors, such as fear of losing a job during the COVID-19 outbreak, exposure to COVID-19 rumours, susceptibility to COVID-19, and movement restrictions were reported to be determinants of depression and anxiety. However, our study found no significant difference in the prevalence of mental disorders before and during the COVID-19 period.

To our knowledge, our study is the first to estimate the pooled prevalence of substance use among international migrant workers, particularly in the Southeast Asia region. Our study found that one in four migrant workers use substances in some form (24.29%) and one in three drank alcohol (30.52%). When analyzing substance use by type, the prevalence of hazardous/risky drinking was low (10.13%), but the prevalence of current drinking and occasional heavy drinking was high at about 38% and 44%, respectively. This suggests that although the prevalence of current drinking tends to be lower in many ASEAN countries compared to high-income countries, the prevalence of unrecorded drinking and occasional heavy drinking is relatively much higher, which should be taken into account in the interpretation [53]. Meanwhile, only a limited number of studies on substances among migrant workers in the region were identified, and some of the included studies are also regarded as of low quality, which may contribute to the large variability in the prevalence estimation. Furthermore, the different assessment tools used can lead to variations in prevalence between studies. Of the included studies on alcohol use, only two studies [18,34] used the AUDIT, a validated instrument for identifying people who drink hazardously or have active alcohol use disorders (including alcohol use disorder or dependence) [54]. In contrast, the other studies assessed alcohol drinking status using a single or a few questions with different reference periods (e.g. a week, a month, or a year), or two did not specify the assessment methods. Tobacco use is also common among migrant workers. The fact that current smokers among the adult population in ASEAN account for 10% of adult smokers worldwide [55] may support our study reporting that 42% of migrant workers used tobacco in some form. By tobacco type, the prevalence of smoking was 30.55%, while that of tobacco (smoking and/or smokeless) was 59.15%. In terms of illicit drugs, of the four included studies on illicit drugs, the pooled prevalence is 1.6%, with the commonly used form being Crystal Meth/Ice/Sketch (10.7%). The prevalence of substance use, particularly alcohol and illicit drugs, seems lower in the region than in other studies. A study among Hispanic migrants in the USA suggested that the overall prevalence of last 12-month drinking, smoking, and illicit drug use was 42.3%, 31.4% and 17.7%, respectively. Male migrants were at a greater risk for illicit drug use in the destination country, compared to pre-departure migrants, and undocumented migrants showed a higher prevalence of illicit drugs, compared to their documented counterparts [56]. Exposure to these behavioral factors, including excessive drinking and smoking, has been shown to contribute to the high incidence of chronic diseases and mortality in Southeast Asian countries [57,58]. Hence, awareness and education about substance use among international migrant workers is imperative. Interestingly, all studies on substance use in our study were conducted only in Thailand. Thailand is a market and transit channel for drug trafficking to third countries, due to Thailand’s geopolitical transportation infrastructure adjacent to the Golden Triangle region (bordering Myanmar, Lao PDR, and Thailand), which is Asia’s largest drug production area. Due to the development of drug production technology and changes in transaction patterns through online and parcel posts, the price of illegal drugs produced in the region continues to fall [59]. Such a situation makes illegal drugs easily accessible to people, including migrants. Because the number of studies on substance use, particularly illicit drugs, was limited, and most studies included in the review were small and employed convenience sampling, more studies with robust sampling methods to represent international migrant workers in ASEAN member countries are warranted. More specific efforts to prevent and treat substance use among immigrants should be made.

In terms of determinants of substance use, only a few studies were included for a narrative synthesis. Men were more likely to use substances than women. Longer years spent working in the country, a history of drinking in the country of origin, and other substance use, like tobacco, were associated with alcohol drinking. Prevalence of multiple substance use, including alcohol and smoking, was high at 20–30% [34]. Migrant workers having friends who used any substance were more likely to practice multiple health-risk behaviours than those who did not have such friends [34]. This may imply that peer support groups are considered an important aspect of the addiction recovery process since they offer unique benefits in engaging populations that are difficult to engage, and a reduction in habitual craving and feelings of guilt [60]. Thus, efforts should be made to create a healthy, drug-free environment, along with peer support services, in various ways, including in-person self-help groups, online support groups, and peer partnerships [60].

Our study showed the high prevalence of mental health conditions and substance use among international migrant workers. This implies that more special efforts from governmental and non-governmental organisations and regional political organisations, with their close collaboration, must be provided to prevent, screen, and manage mental health and substance use problems among the international migrant population in the region. For example, training on awareness and knowledge of mental health and substance use, as well as their health impacts, should be provided to migrant workers and their managers during the initial period of migration. Regular workplace consultations or monitoring (monthly or as needed) should also be conducted. In addition, multilingual psychological counselling and peer support should be provided to address the mental health issues and substance use of migrant workers in the community, as well as the establishment of community-based focal points to provide emergency support in the event of a sudden emergence of such problems. The ASEAN Health Cooperation has identified 20 health priorities, including mental health and substances such as alcohol, tobacco, and illicit drugs, with the aim of improving the health of people in ASEAN countries through shared goals, strategies, and programs in the health sector [55,61]. Most LMIC countries in ASEAN have national mental health policies. Still, most countries face multiple challenges, including a lack of resources to fund mental health services and a shortage of mental health personnel [61–63]. In Thailand, Malaysia, and Singapore, which host large numbers of international migrant workers from LMICs, there is an urgent need to implement workplace- and community-based education and awareness programmes on mental health and substance abuse for international migrant workers. In addition, few systematic reviews and meta-analyses have assessed the prevalence and determinants of mental illness and substance use exclusively among international migrant workers in the ASEAN countries. In particular, to the best of our knowledge, this is the first study to conduct a meta-analysis on substance use among international migrant workers. The study’s findings may serve as a reference for policymakers, authorities, and employers in developing preventive strategies for migrant workers. Since the limited number of studies in the region and the use of convenience sampling were included in the meta-analysis, this review suggests that future studies on mental health and substance use should employ probability sampling methods and have larger sample sizes.

This study has several limitations that should be noted. Firstly, since all the included studies employed a cross-sectional design, no causal relationship can be established. Moreover, the use of non-probability sampling and the exclusion of irregular migrant workers in most studies limit the generalisability of their findings to represent the entire migrant worker population. The use of non-representative and small samples suggests a high risk of bias. Next, the high heterogeneity of tools across studies and the reporting of some risk factors in only one study make it difficult to generate a pooled effect size, requiring careful consideration when interpreting the results of this review. In addition, this study searched and extracted articles only from selected online databases, excluding journal articles written in languages other than English, gray reports, and databases such as PsycINFO, which may have resulted in the exclusion of some studies that met the inclusion criteria, potentially compromising the representativeness of the entire study. Due to the limited number of studies on other outcome variables, sensitivity analyses were conducted only for depression. Our study listed related factors narratively; however, it is expected that future research on these topics will enhance the ability to measure the effect size of moderator variables on mental health and substance use through meta-regression analysis.

## Conclusion

This systematic review and meta-analysis of international migrant workers in ASEAN countries found a high prevalence of mental health conditions and substance use, suggesting that screening and treatment services should be provided through closer collaboration between governments and non-governmental organisations.

## Data Availability

The data utilised in this review were derived from publicly available sources and literature.
